# Two-way NxP fertilisation experiment on barley (*Hordeum vulgare*) reveals shift from additive to synergistic N-P interactions at critical phosphorus fertilisation level

**DOI:** 10.3389/fpls.2024.1346729

**Published:** 2024-03-05

**Authors:** Jessica Clayton, Kathleen Lemanski, Marcel Dominik Solbach, Vicky M. Temperton, Michael Bonkowski

**Affiliations:** ^1^ Terrestrial Ecology, Institute of Zoology, Cluster of Excellence on Plant Sciences (CEPLAS), University of Cologne, Cologne, Germany; ^2^ Institute of Ecology, Faculty of Sustainability, Leuphana University Lüneburg, Lüneburg, Germany

**Keywords:** synergistic effects, N-P interactions, barley, nutrient limitation, shoot:root ratio, fertilisation, stoichiometry, critical P level

## Abstract

In a pot experiment, we investigated synergistic interaction of N and P fertilisation on barley biomass (*Hordeum vulgare*) on both shoot and root level with the aim to determine whether N-P interaction would be the same for all levels of N and P fertilisation. We further aimed to determine whether there was a critical level of N and/or P fertilisation rate, above which, a decrease in resource allocation to roots (as nutrient availability increased) could be demonstrated. Barley plants were grown from seed on a nutrient poor substrate and subjected to a two-way NxP fertilisation gradient using a modified Hoagland fertilisation solution. We observed N-P interactions in shoot and root biomass, and N and P use-efficiencies. A synergistic response in biomass was observed only above a critical level of P fertilisation when P was not limiting growth. Furthermore, we found that the same incremental increase in N:P ratio of applied fertiliser elicited different responses in shoot and root biomass depending on P treatment and concluded that barley plants were less able to cope with increasing stoichiometric imbalance when P was deficient. We provide, for the first time, stoichiometric evidence that critical levels for synergistic interactions between N-P may exist in crop plants.

## Introduction

Nitrogen (N) and phosphorus (P) are the two most important macronutrients that limit plant growth (Elser et al., 2007; Sterner and Elser, 2008). Both nutrients can limit growth since both nitrogen and phosphorus are needed for core metabolic activities (Elser et al., 2000). Classic models of nutrient limitation are based on Liebig’s Law of the Minimum. The law states that the scarcest nutrient (in relation to the organisms’ requirements) limits growth and once the requirements for this nutrient have been met, the next scarcest nutrient will become the new limiting nutrient (Ågren et al., 2012). Yet in the last two decades new models have emerged, in which nutrient limitation is a product of co-limitation or multiple-limitation to multiple nutrients simultaneously, whereby nutrients are required together in some optimum ratio (Saito et al., 2008; Harpole et al., 2011; Ågren et al., 2012).

Nutrients which are co-limiting may also interact (Elser et al., 2007). These so-called nutrient interactions can be either synergistic; the combined effect on growth is greater than the sum of the individual effects of each nutrient, or negative; the combined effect is less than the sum of the individual effects (Davidson and Howarth, 2007). Nitrogen and phosphorus as key nutrients have been extensively studied in agroecosystems, yet despite their generally synergistic effects on cereal crop yield and nutrient use efficiency, interaction effects between N and P applications have been far less extensively studied (Elser et al., 2007; Rietra et al., 2017; Duncan et al., 2018a; Duncan et al., 2018b; van Duijnen et al., 2021).

Yet, to fully understand N-P interactions in crop yields and nutrient use efficiencies, we must first look underground and see how plants invest in above- and belowground tissues across different N and P availabilities, thus considering scenarios where nutrient supply is neither optimum nor balanced. Plants are extremely adaptable to changing nutrient conditions and are able to optimally redistribute resources among the tissues where they are required (Wilson, 1988; Hilbert, 1990; van der Werf and Nagel, 1996; Temperton et al., 2003; Sadras, 2006). When nutrients are scarce, plants increase investment into root biomass, causing a decrease in shoot:root ratio (Robinson, 1994; van der Werf and Nagel, 1996; Scheible et al., 1997; Hermans et al., 2006). However, this optimisation can come with a cost to aboveground productivity leading to a shoot-root trade off (Li et al., 2010; Kim and Li, 2016).

Adaptations such as changes in root architecture in response to variation in nutrient availability may allow for optimised nutrient uptake. In heterogeneous supply of N and P, localised increases in lateral roots have been observed in the areas of higher nutrient concentration (Drew et al., 1973; Drew, 1975; Drew and Saker, 1978) as well as overall increased nutrient uptake flux by roots to compensate for the non-uniform supply (Robinson, 1994). Moreover, Kumar et al. (2019) found that root diameter corelated with colonisation of arbuscular mycorrhizal fungi for optimised P uptake. There are also differing effects of N and P limitation on root architecture. Kumar et al. (2020) found that plant roots foraged in deeper layers when N was the limiting factor, but explored the topsoil when P was limiting. In a complementary experiment where N and P were added at different timepoints in the growing phase, delayed N addition had a stronger negative effect on biomass than delayed P (van Duijnen et al., 2021).

Although there is plenty of evidence of the underground responses to changes in N and P availability independently, little is known about the effects of an N-P interaction on root growth, i.e., due to changes in both N and P. Duncan et al. (2018b) showed that there was a positive interaction between N and P on root growth as well as nitrogen use efficiency and nitrogen retention in wheat. It is however not so well understood how nutrient interactions may shape the allocation of nutrients to shoots and roots. The aboveground studies of N-P interaction on cereals in most cases have a simple experimental design with few combinations of low vs high N and P fertilisation rates (Prystupa et al., 2003; Prystupa et al., 2004) or fewer P rates compared to N (Michaelson et al., 1982; Tigre, 2014). Fewer studies have investigated nutrient interactions across a high number of fertilisation rates. Duncan et al. (2018a) identified 11 fertilisation experiments on wheat in which the design had at least two N fertilisation rates and multiple P fertilisation rates. Through investigating N-P interaction on both the shoot and root level, it may be possible to gain insight into shoot-root trade-offs and give answers to the following questions: At what combined level of N and P do we see shifts to decreased root investment with increased N and P levels? What happens when we subject plants to gradients of N and P in magnitudes ranging from inadequate to adequate supply to the plant’s requirements?

In a pot experiment, we investigated the N-P interaction on shoot and root biomass of barley (*Hordeum vulgare*) along a two-way N and P fertilisation gradient. We subjected the barley plants to varying degrees of N and P deprivation using a modified Hoagland solution (Hoagland and Arnon, 1950). We chose 6 levels of N and 6 levels of P fertilisation, resulting in 36 N and P combinations and N:P ratios ranging 2-120. This covers a wide range of different ratios and includes the narrow range of 4-6, the range in which Sadras (2006) found attained the maximum yield in over 40% of the 1500+ crops he assessed.

We hypothesise that there will be positive interactions between N and P fertilisation treatments due to N-P colimitation. Due to trade-offs in plant’s below vs. aboveground allocation, we expect to find a critical stoichiometric threshold as nutrient provision increases, where plant investments in root biomass would be reduced in favour of shoot biomass. Finally, we hypothesise that changes in barley biomass yield per unit of applied N or P fertilizer (i.e. nitrogen and phosphorus use efficiency) show similar patterns (i.e. would be statistically independent) along the fertilization gradient.

To test these hypotheses, we set up a pot experiment with an N-P gradient using extremely low-nutrient soils from a mining site, where we expected microbial communities to be very limited.

## Materials and methods

### Preparation of seeds for germination and pot preparation

Barley (*Hordeum vulgare*) seeds were sterilized for 1 minute with 70% ethanol under vacuum and for a further 2 minutes with chlorine bleach (5% sodium hypochlorite) and finally washed with sterile water. Under sterile conditions, single seeds were planted into small plastic tubes (< 5 ml volume) with sterile sand and watered as required (**Supplementary Figure S1A**).

Pots with volume of ~2 l were prepared with a substrate of loess soil mixed with sand in ratio 1:4 (**Supplementary Figure S1B**). There were 36 fertilisation treatments each replicated 8 times, giving 288 pots in total. A nutrient-poor silt loess soil (Cambisol), devoid of soil organic matter, was collected from deep sandy layers at a lignite mining site in Jackerath (North Rhine-Westphalia Germany), at a depth of 5 to 12 m. The soil was oven dried overnight at 60°C to kill spores of arbuscular mycorrhiza and any potential soil fauna (Endlweber and Scheu, 2006). Three seedlings per pot were planted and were placed in a temperature-controlled greenhouse. The plants were allowed to grow for 8 weeks in the greenhouse, were watered as necessary and supplied with fertiliser as per the experimental set up for the fertilisation gradient. After every fertilisation, plants were randomly repositioned to minimize edge effects.

### Two-way N and P fertilisation gradient

**Figure 1:**
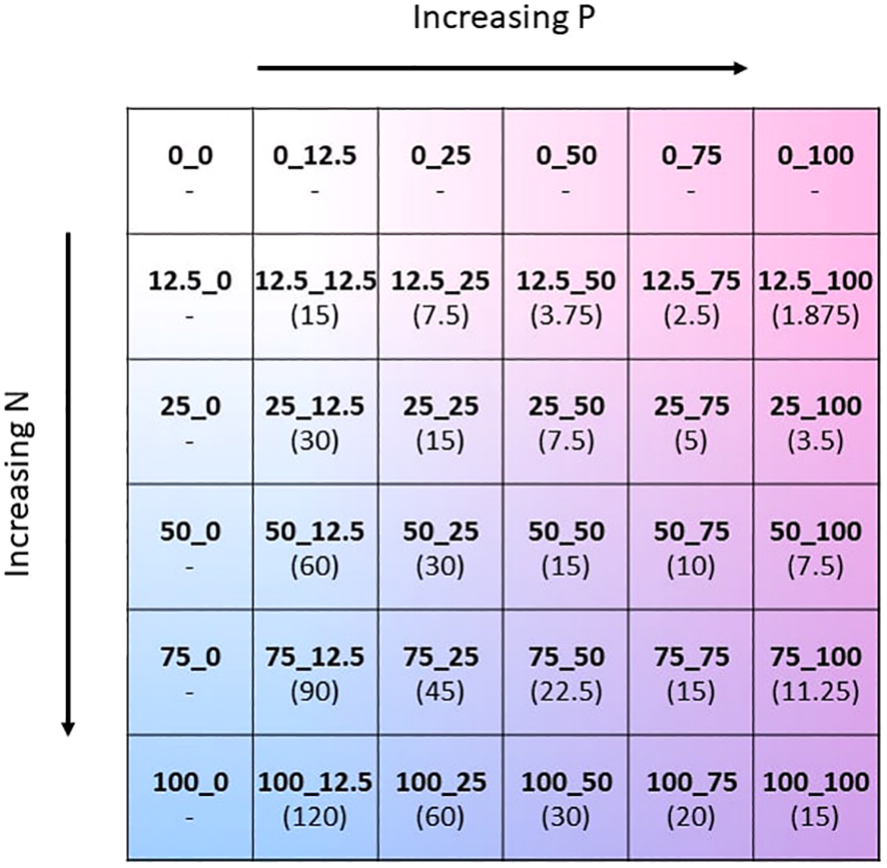
Schematic of the two-way NxP fertilisation gradient depicted as a matrix. The values 0, 12.5, 25, 50, 75, and 100 correspond to % of the original N or P concentration in the Hoagland solution. Absolute molar concentrations of N and P for each treatment can be found in **Supplementary Table S2** of the **Supplementary Material**. N:P ratio of the fertiliser mixture of each treatment in brackets.

The two-way fertilisation gradient was created by modifying both the N and P concentration of the Hoagland solution (Hoagland and Arnon, 1950), whilst keeping all other nutrient concentrations unchanged. The concentration of N and P containing substrates were altered to give separately 6 concentration levels: 0, 12.5, 25, 50, 75, and 100% of the original Hoagland solution. This gave 36 unique combinations of N and P concentrations (**Figure 1**). In order to reduce N and P in Hoagland solution, the nutrient salts KH_2_PO_4,_ KNO_3_, and Ca(NO_3_)_2_ were reduced accordingly. The missing potassium and calcium as a result of reduction of the nutrient salts were replaced by the addition of KCl and CaCl. The exact formulation of the modified Hoagland solution for each of the 36 treatments is found in the **Supplementary Material** (**Supplementary Table S1**). Throughout the growing period the amount of solution applied to the plants gradually increased in accordance with the plant’s requirements. Fertiliser solution was applied once weekly and summed to a total of 500 ml of solution applied per pot throughout the experiment. To avoid leakage of fertiliser, the pots were not watered on the same day that fertiliser was applied.

### Harvesting and analysis of plant material

A detailed record of plant height and number of plants per pot was taken every week. After sufficient growth, the barley plants were harvested at 48 days and roots, shoots and ears (if present) were separated and dried at 60°C. Roots were washed before drying. The dry weight of the biomass was determined for both roots and shoots. Total carbon (C) and total N contents were determined in both shoot and root tissue using a Flash 2000 Organic Elemental Analyzer (Thermo Scientific).

In order to determine whether there was a synergistic or negative effect of the combined N and P treatments, we calculated the expected yield (Y_NP_) for biomass, total C and total N in both shoots and roots (Equation 1) as per Rietra et al. (2017). Where Y_N_ is the mean yield of the N only treatments (when P = 0 and N > 0), Y_P_ is the mean yield of P only treatments (when N = 0 and P > 0), Y_0_ is the control (when N = 0 and P = 0), and Y_NP_ is the expected yield of N and P combined (Equation 1). We used the mean, as there was little within group variation of the N = 0 and P = 0 treatments, i.e., there was no effect of P on N = 0 treatments and no effect of N on P = 0 treatments. The error on the expected yield (ΔY_NP_) was estimated using the standard errors ΔY_N_, ΔY_P_, and ΔY_0_ of their respective means (Equation 2). When the observed yield was greater than Y_NP_, there was a synergistic response. Observations below Y_NP_ indicated negative responses, and observations within Y_NP_ ± ΔY_NP_ indicated additive responses.


(1) $$\text{Expected yield}:\;\frac{Y_{NP}}{Y_{0}}=\;\frac{Y_{N}}{Y_{0}}\times \frac{Y_{P}}{Y_{0}}$$

(2) $$\text{Estimated error }\Delta Y_{NP}=\;Y_{NP}\times \sqrt{{\left(\frac{\Delta Y_{N}}{Y_{N}}\right)}^{2}+{\left(\frac{\Delta Y_{P}}{Y_{P}}\right)}^{2}+{\left(\frac{\Delta Y_{0}}{Y_{0}}\right)}^{2}}$$

Shoot:root (SR) ratios were calculated for biomass, total C and total N. For example, SR-Biomass = shoot biomass (g)/root biomass (g), SR-Total C = Shoot total C/Root total C, SR-Total N = Shoot total N/Root total N. Nitrogen-use efficiency (NUE) and phosphorus use efficiency (PUE) were calculated as the biomass per applied N and P fertilisation for roots and shoots respectively. For example, Shoot NUE = shoot biomass (g)/applied N fertilisation (mmol) and Shoot PUE = shoot biomass (g)/applied P fertilisation (mmol).

## Data handling and statistics

To avoid bias in the data, only pots which contained all 3 barley plants at the end of the growing period were included in the analysis. There were 66 pots which did not meet this criterion due to failure to grow or predation by rodents in the greenhouse.

To get an overview of the whole dataset (total 222 pots) we applied Manova models using the predictor variables N and P fertiliser treatments and log_10_(N:P ratio). From each significant Manova model, Anova statistics were extracted to see the effect of the predictor variables (e.g. N and P fertilisation) on each individual response variable (i.e. protected Anova (Scheiner et al., 2001)). The results of the Manova and Anova models can be seen in detail in the **Supplementary Material** (**Supplementary Tables S2–S4**).

All analyses and data handling were conducted in R (version 3.5.0) with the use of packages: *dplyr* (Wickham et al., 2018b), *tidyr* (Wickham et al., 2018c), *broom* (Robinson et al., 2021), and *purrr* (Henry and Wickham, 2019). Figures were produced using the R packages *ggplot2* (Wickham et al., 2018a), *ggpubr* (Kassambara, 2020), and *RColorBrewer* (Neuwirth, 2014). Statistical model validation was carried out as per Zuur et al. (2010). Significance levels reported throughout the manuscript are as follows: p > 0.05 (ns), p ≤ 0.05 (*), p ≤ 0.01 (**), and p ≤ 0.001 (***).

## Results

### Shoot and root biomass, total C and total N

**Figure 2:**
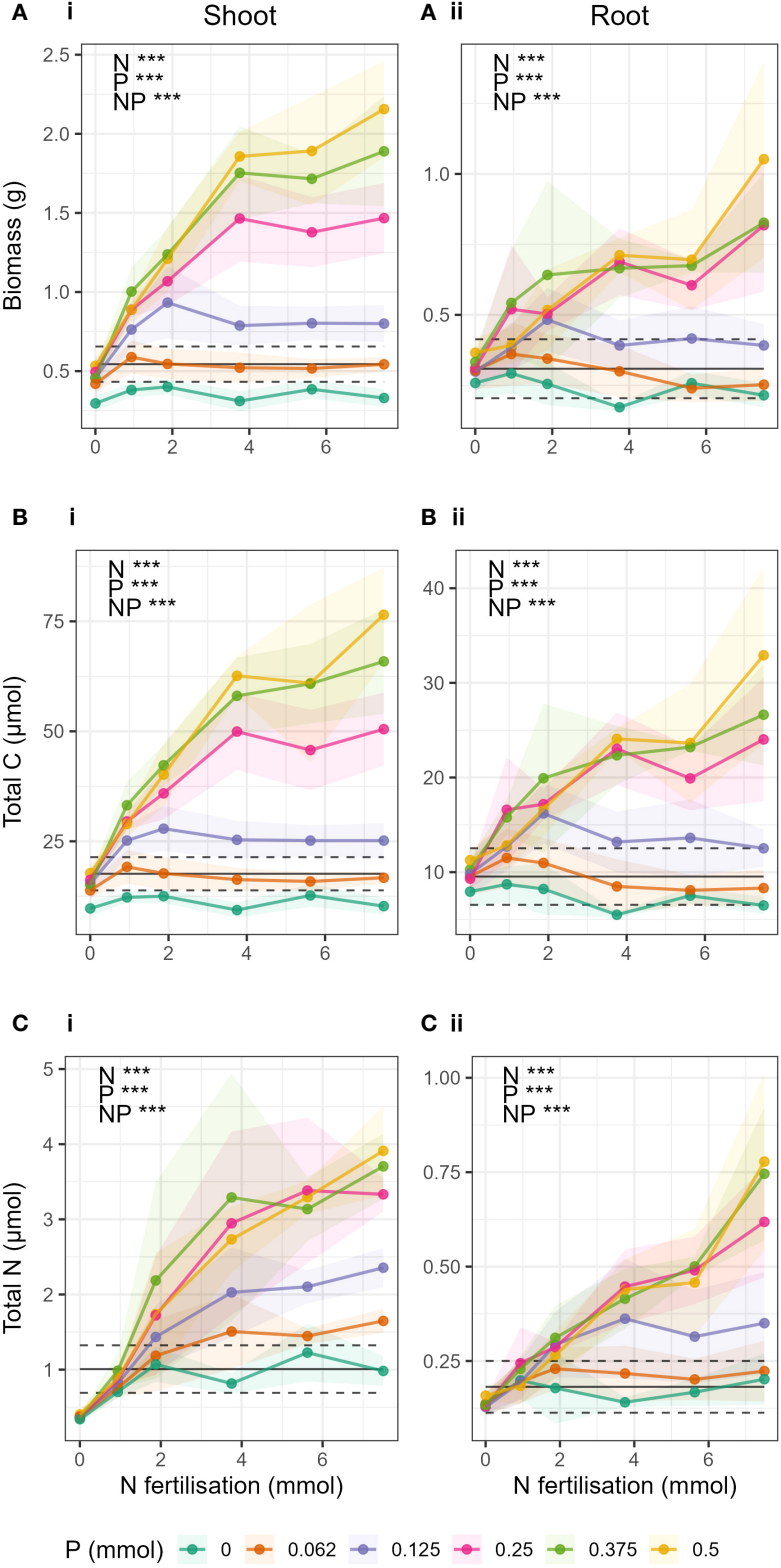
Change in **(A)** biomass, **(B)** total C and **(C)** total N in respect to N fertilisation (x-axis) and P fertilisation (coloured lines) in i) shoots and ii) roots. Points show mean value for treatment and shaded area around the lines drawn by connecting the points ± 1 standard deviation from the mean. Black lines indicate the expected yield (Y_NP_) if N and P were applied separately. The dashed black lines give ± ΔY_NP_ (the estimated error on Y_NP_, see methods). When the observed yield was greater than Y_NP_, there was a synergistic response. Observations below Y_NP_ indicated negative responses, and observations within Y_NP_ ± ΔY_NP_ indicated additive responses.

The gradient in N and P fertiliser caused significant change across nearly all response variables (**Figure 2**), and significant interactions between N and P fertiliser treatments and plant tissue (shoot/root) were found (Suppl. Manova model **Supplementary Tables S2, S4, S7, S9**).

As expected, total barley biomass, total C and total N in shoots and roots significantly increased both with increasing N and increasing P fertilisation, and there was a significant N-P interaction (**Supplementary Table S3**). The interaction could be clearly seen in the difference in response of biomass, total C, and total N to N fertilisation at different levels of P fertilisation (**Figure 2**). When P was low (P ≤ 0.25 mmol), biomass and total C in shoots and roots (**Figures 2A, B**) did not increase significantly with increasing N fertilisation, and there was generally no response to N addition. Only in treatments where P > 0.25 mmol were there strong effects of N fertiliser, where increases in N fertilisation led to large increases in biomass and total C. There was also little or no difference due to the effect of increasing P in treatments where P > 0.25 mmol.

Barley total biomass, total C and total N in shoots and roots showed synergistic responses to combined N and P fertilization compared to yields expected if N and P were applied separately (**Figure 2** black lines) when P > 0.25 mmol and N > 0 (**Figures 2A**-i, **2B**-i, **2C**-ii). For shoot total N, the synergistic response occurred at a lower P threshold, in treatments where P > 0.062 mmol. In biomass and total C of shoots, there were negative responses to increases in N fertiliser (i.e., yield was less than expected yield when N and P were applied separately) when P was absent (P = 0 treatments), and additive responses (yield was statistically the same as expected yield) when P = 0.062 mmol. This was the same for roots, except that the P = 0 treatment also showed an additive response to increases in N fertilisation. For total N, there were no negative interactions, only additive responses to increasing N fertilisation in treatments where P< 0.25 mmol.

In biomass and total C where P = 0.25 mmol, the responses to N addition were mostly additive, except for the treatments where N and P Hoagland proportions were balanced (e.g. N_P 25_25, corresponding to 0.25 mmol N and 0.125 mmol P), where a synergistic response was seen.

In the treatments where P ≤ 0.25 mmol, peaks in total biomass and total C were observed at each P level (**Figures 2A, B**). The peaks corresponded to the treatments where the Hoagland proportions of N and P were equal (e.g. 25_25, 50_50, etc). However, in treatments where P > 0.25 mmol it was not possible to determine if biomass had peaked. In roots (**Figure 2B**), the peaks occurred only in N_P treatments 12.5_12.5 and 25_25.

#### Responses to fertiliser N:P ratio

**Figure 3:**
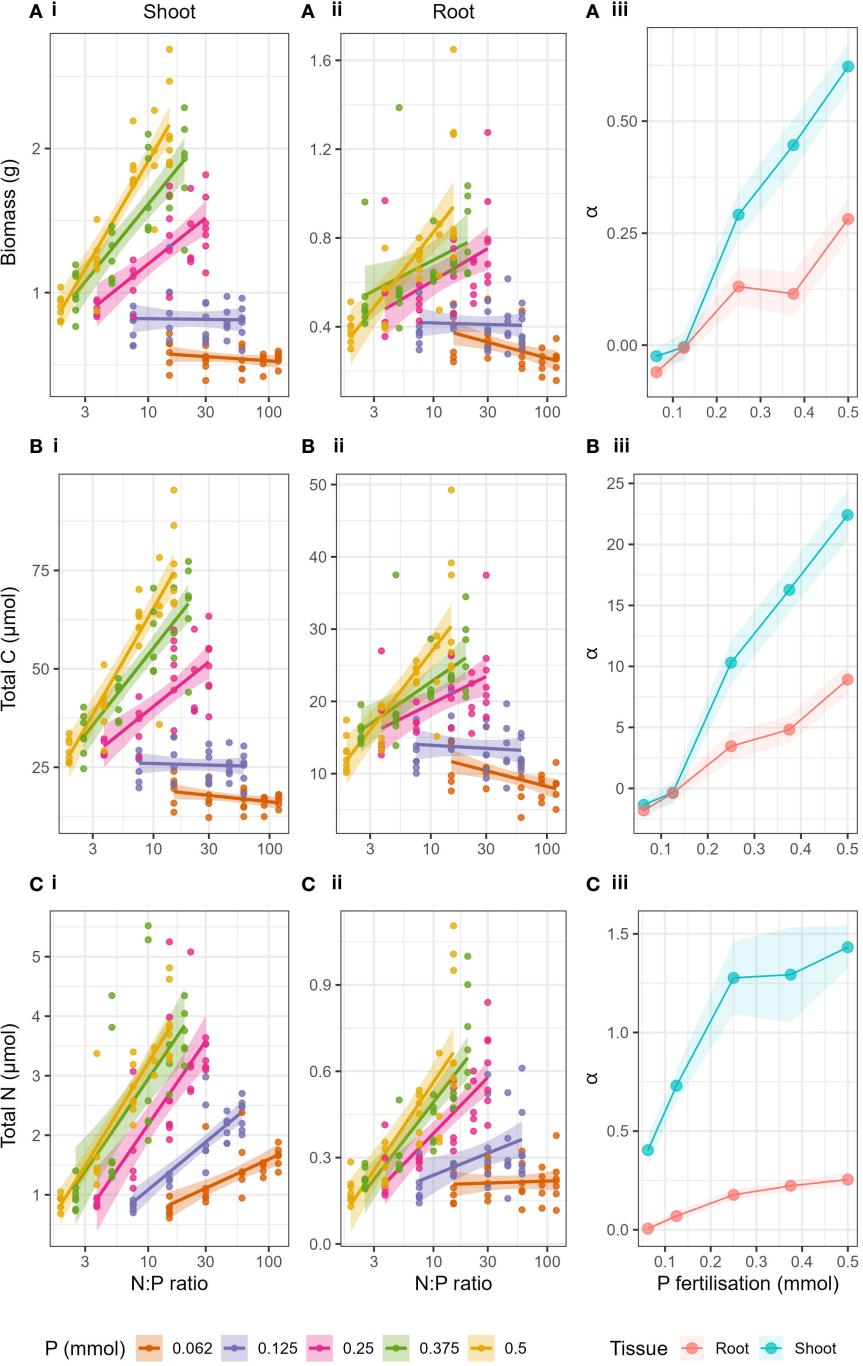
Biomass **(A)**, Total C **(B)** and Total N **(C)** with changing N:P ratio of the applied fertiliser (log transformed x axis) and P fertilisation (lines) for i) shoots and ii) roots. Panel iii) show the slopes (α) of the linear regressions of each response variable with N:P ratio for both roots and shoots with respect to P fertilisation. Shaded areas around lines represent the standard error.

The shift in the relationship of the response variables to N at a critical P value was more clearly seen when plotted against log transformed N:P ratio of the applied fertiliser treatment (**Figure 3**). When looking at the P levels independently, increases in biomass, total C and total N with increasing N:P ratio were caused by increases in N fertilisation (as P was constant) and the resulting relationships were linear (**Supplementary Table S4**). Biomass, total C and total N in shoots and roots increased with increasing N:P ratio when P > 0.25 mmol (**Supplementary Table S4**). However, when P ≤ 0.25 mmol, there was either no effect of increased N:P ratio or a negative effect. The linear dependency made it suitable to compare slopes (α) of the relationships for each P level (**Figures 3A**-iii, **B**-iii, **C**-iii). The slopes (α) of the regressions of biomass, total C and total N to log_10_(N:P), in both roots and shoots, increased with increasing P fertilisation. Here we could show very clearly that as P level increased, the effect of N:P ratio (or simply N) on the response variables increased. Particularly we see a great shift in the slopes (α) between P = 0.125 mmol and P = 0.25 mmol in both shoots and roots. The effect of increasing P was strongest in shoots compared to roots (see full Anova and regression statistics, **Supplementary Tables S5, S6**).

### Shoot:root ratios

**Figure 4:**
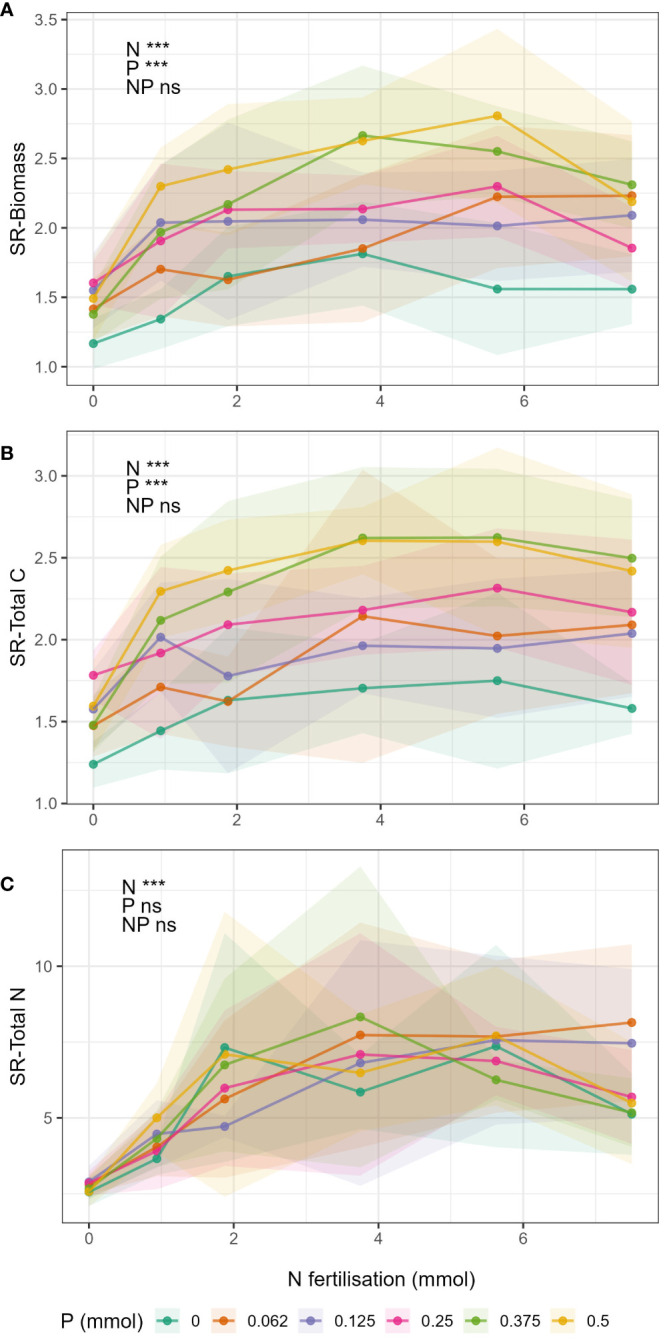
Change in shoot:root ratios in respect to N fertilisation (x-axis) and P fertilisation (lines) for the different response variables **(A)** biomass, **(B)** total C, and **(C)** total N. Points show mean value for treatment and shaded area around the lines drawn by connecting the points ± 1 standard deviation from the mean.

Shoot:root ratio of biomass and total C (SR-Biomass and SR-Total C, **Figures 4A, B** respectively) increased with increasing N and P fertilisation, but there were no significant N-P interactions (**Supplementary Table S8**). SR-Total N ratio (**Figure 4C**) increased with increasing N fertiliser but there was no effect of P, nor a significant N-P interaction (**Supplementary Table S8**).

### Nutrient use efficiencies

**Figure 5:**
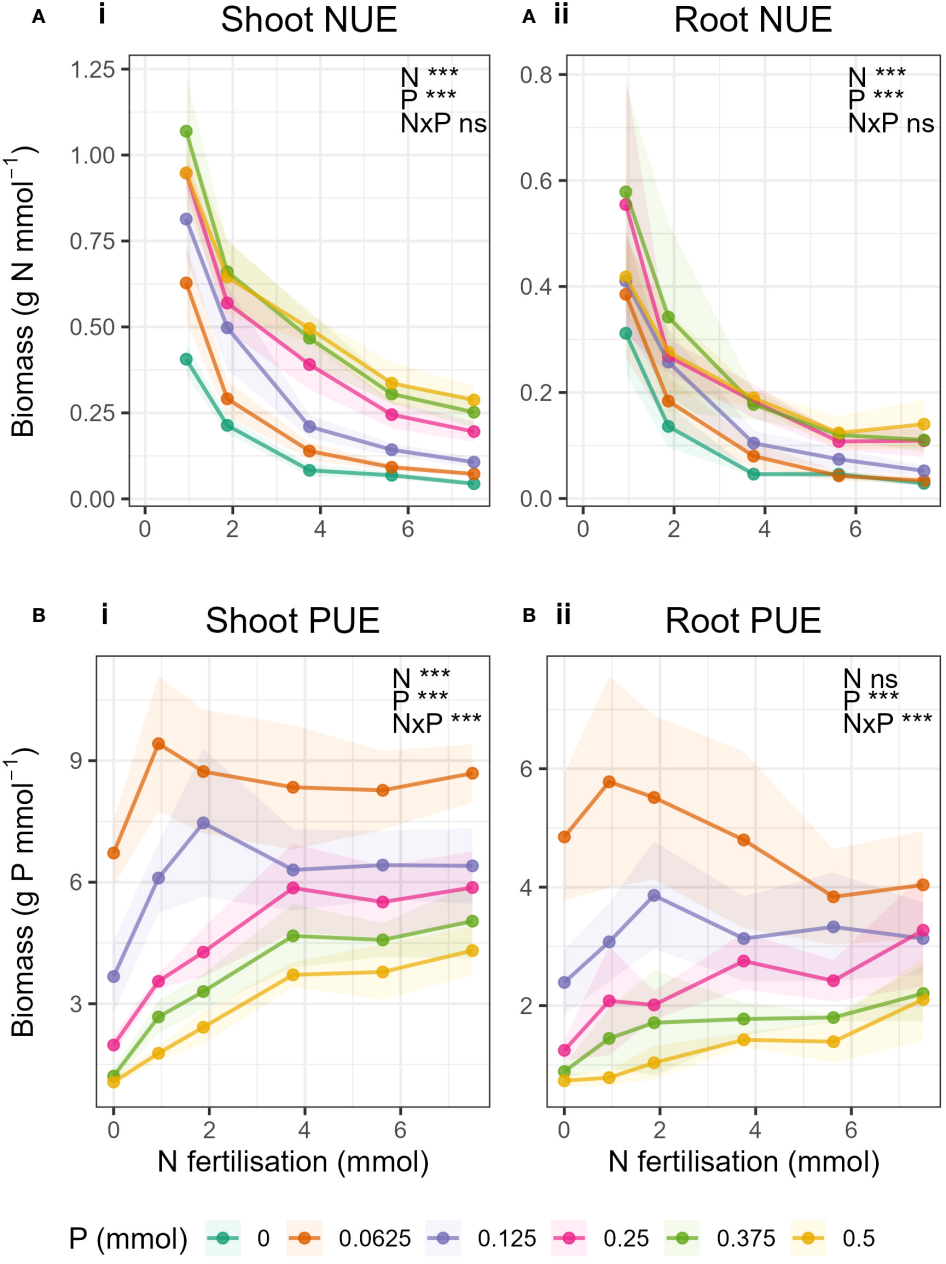
Nutrient use efficiency, **(A)** NUE and **(B)** PUE in in i) shoot- and ii) root biomass with changing N fertilisation (x-axis) and P fertilisation (coloured lines). Nutrient use efficiency was calculated as the biomass (g) per mmol nutrient applied. Dots represent the mean and shaded areas correspond to ± 1 standard deviation.

Generally, NUE decreased with increasing N fertilisation and increased with increasing P fertilisation (**Figures 5A**-i, **5A**-ii). Similarly, PUE decreased with increasing P fertilisation and increased with increasing N fertilisation (**Figures 5B**-i, **5B**-ii). No N-P interaction was observed in NUE in either shoots or roots, i.e., the rate of decrease in NUE due to increased N did not change with increasing P, but the magnitude of the NUE increased with increasing P (**Supplementary Tables S9, S10**). Whereas with PUE, there was an interaction in both roots and shoot. PUE in shoots generally increased with increasing N, but, unlike NUE, the relationship was not the same for all P levels. When P was high (P ≥ 0.25 mmol) PUE increased with increasing N but only up to N = 3.75 mmol, and above which was constant. In the low P treatments, initially PUE increased strongly with increasing N but peaked and then decreased again with increasing N. There was no overall statistical effect of N on PUE in roots.

## Discussion

### Synergistic N-P interactions and critical level of P

Unsurprisingly, the treatments with low N and low P fertilisation resulted in low yields of biomass, total C and total N, and it was to be expected that there would be increases in yields with increasing N and P. Yet what is interesting in our data, is that we saw that the rate of increase in biomass, total C and total N due to increasing N fertilisation was not the same for all levels of P fertilisation. In fact, we saw synergistic interactions between N and P only above a critical threshold of P fertilisation, in both shoots and roots.

The critical level of P observed for biomass and total C in this study can be interpreted as the threshold between two states of growth limitation by P as characterised by differing responses of yield to changes in N availability. The state where P was growth limiting (P ≤ 0.25 mmol), biomass did not respond to increasing N. Whereas above this critical level, in a state where P is not growth limiting (treatments P > 0.25 mmol), further increases in P above 0.25 mmol had no or very minimal effect on biomass yield and conversely increasing N had a strong positive effect on yield (**Figure 2**).

The synergistic effect of N and P occurred in biomass, total C and total N in both shoots and roots (**Figure 2**), whereby the yield when N and P were applied together was greater than the expected yield if N and P were applied separately. This result further confirms the effect as seen in various studies (Zubillaga et al., 2002; Tigre, 2014; Rietra et al., 2017; Duncan et al., 2018a; Kumar et al., 2020). Yet crucially in our results, a synergistic effect was only observed at P > 0.25 mmol. When P was less than or equal to the critical level, there was an additive effect; at this level of P, the effect of N and P applied together was the same as the expected yield. Interestingly, the critical value of P, which determined a shift between synergistic and negative N-P interaction, was lower in shoot total N (P = 0.062 mmol) compared to the other variables (**Figures 2C**-i). This shows that only the smallest amount of P aided N acquisition in shoots and that this effect was very large, which corroborates with the finding in another wheat study that tested N-P gradient effects (Duncan et al., 2018b).

### Effect of widening N:P ratio

The effect of the critical P value could be explained due to the ever increasing stoichiometric imbalance caused by the widening of the N:P ratios as P decreased and N increased (Güsewell, 2005; Ågren et al., 2012; Yan et al., 2015). When the ratio of the nutrients supplied deviates from the optimum, an imbalance is created causing a relative excess of one nutrient to a relative deficiency in others (Elser et al., 2007; Reich, 2017). This can also lead to feedbacks, i.e., as the demand for one nutrient is satisfied, this can lead to a higher demand for other nutrients (Reich, 2017). The range of N:P ratios of the highest P level (P = 0.5 mmol) in our study was 1.9 – 15, whereas the range of N:P ratios of the lowest (P = 0.062 mmol) was 15-120 (**Supplementary Table S11**). This fits well with the findings of Sadras (2006) whereby crop species had an N:P ratio of between 4 and 6 when managing optimal yield. Our plants only saw such an N:P range when supplied with enough P (P ≥ 0.25 mmol), but went higher than this range when supplied with lower than 0.25 mmol P.

Breakpoints in foliar N:P ratio have been shown to exist which determine nutrient limitation, whereby foliar N:P > 16 is indicative of P limitation and N:P< 14 is indicative of N limitation (Aerts, 1996; Koerselman and Meuleman, 1996). However, we did not see a breakpoint as such in our results, as ranges of fertiliser N:P ratios for each P level were not discrete but overlapped (**Figure 3**). Despite this we still clearly saw a threshold level of P due to the large shifts in slopes between P treatments P > 0.25 mmol and P ≤ 0.25 mmol (**Figure 3**), most notably in biomass and total C. The differences between slopes were less extreme in total N but slopes still increased with increasing P level. Ågren et al. (2012) discussed the possibility that shifts in nutrient limitation can occur over a range of N:P ratios rather than a discrete deviation from a fixed optimum ratio, this too could explain our result of synergistic interactions occurring across a range of N:P ratios, albeit when N:P ratio was low.

We can speculate further that the plants in P deficiency were less equipped to cope with widening N:P imbalance compared to when N was limiting, and therefore a positive effect of N was not observed when P was limiting. A similar result was also observed in an NxP fertilisation experiment on *Arabidopsis thaliana* with 3 levels N and P fertilisation respectively (Yan et al., 2015), whereby growth rate of green leaves increased with fertiliser N:P ratio in N limited conditions, but decreased when P was limited (at the lowest P fertilisation level).

Our data show that changes in N:P ratio had differing effects depending on the absolute P availability (**Figure 3**). This could mean that if P is deficient, plants may not cope well with small changes in N:P ratio. Whereas plants may cope better with the same change in N:P ratio when P is less limiting or more available. This highlights that when nutrient availability is low, it is much more important to have a balanced supply of N and P compared to when nutrient availability is high. This has wider implications for nutrient cycling as nutrient imbalance in plants has been shown to have negative feedback on soil organic matter formation (Ding et al., 2021). One solution to reduce fertiliser waste could be to reduce absolute fertiliser application, but we demonstrate here that it should be done carefully, in balance with other nutrients, so as not to create a dangerous imbalance at very low nutrient levels.

### Effect of balanced N:P ratios

When looking at the response curves for biomass, total C and total N for the individual P levels (**Figure 2**) we generally saw peaks in the curves where N and P levels were balanced (e.g. 25% N and 25% P, etc, of original Hoagland solution) with N:P ratio of 15. But interestingly, we saw this effect only in the treatments equal to or below the critical P level, where there was either a negative or additive interaction. The curves of the higher P levels did not appear to saturate unlike in the lower P levels, therefore it was not possible to ascertain whether the maximum responses had been reached. It is well accepted that balanced application of nutrients results in the most positive response (Ericsson, 1995; Güsewell, 2004; Knecht and Goransson, 2004), yet our data show that this was only the case when P was limiting. This could mean that above the critical level, where P was not limiting, balanced application was less important and increases in N generally had positive effects. Whereas when P was limiting, the best-case scenario for the plant was to receive balanced nutrient supply, as increased N supply led to large stoichiometric imbalance (Güsewell, 2004; Ågren et al., 2012). Furthermore, in the treatments where P was equal to the critical value (0.25 mmol), there was generally an additive effect of N and P, except for when both N and P levels were balanced (25_25). Here we saw, in this instance only, that the effect of N and P was synergistic. But soon as N increased again, the effect was lost and returned to an additive response. Again, this is probably due to the inability of the plant to react well to changes in N:P ratios when P was limiting.

A further effect of balanced supply of nutrients was demonstrated in the phosphorus use efficiency (PUE). When P was limiting (P ≤ 0.25 mmol), PUE peaked when N and P supply were balanced, but levelled off as N:P ratio widened (**Figures 5B**-i, **B**-ii). This further illustrates the importance of a balanced supply of nutrients when P was low.

Wide N:P ratio of plant tissue has been shown to result in reduced growth rate (Cernusak et al., 2010; Yan et al., 2015), but there is much less literature on the direct effect of applied nutrients with varying N:P ratio on plant growth. Some of the results here differ from barley root responses in experiments where N-P fertilisation was applied at different stages (van Duijnen et al., 2021) or when N:P stoichiometry of fertilisation was less varied (Kumar et al., 2020). In the latter, Kumar et al. (2020) found an expected linear increase in shoot biomass when moving from low N/low P to high N/high P with the other factors creating outcomes positioned in between (low N/high P; high N/low P). In contrast, having N applied late was far more detrimental than adding P late (van Duijnen et al., 2021), which does not confirm our findings here. The more detailed multi-combination nature of the experimental design in our study allows for a more detailed and mechanistic assessment of the interaction effects of the N and P supply.

### Shoot to root allocation

As in previous results, we expected to see an interaction between N and P in the shoot:root ratios and that there would be a critical value for N and/or P where there would be a clear shift from investment in roots (low shoot:root ratio) to investment in shoots (high shoot:root ratio). However, we did not observe this in our results.

Shoot:root ratios of biomass (SR-biomass), total C (SR-total C) and total N (SR-total N) increased with increasing N and P (**Figure 4**). Or phrased differently, decreases in N and P caused decreases in shoot:root ratio, meaning that there was relative increase in investment into root mass when nutrients supply was low (Wilson, 1988; Hilbert, 1990; van der Werf and Nagel, 1996; Ågren and Franklin, 2003). SR-biomass and SR-total C were significantly affected by both N and P, but interestingly there was no N-P interaction. The effect of N on SR-biomass and SR-total C did not change with changing P, this is contrary to the previous results and to our hypothesis. Moreover, SR-total N was only affected by N and not P. Therefore, P did not have an effect on above-belowground allocation of N in plant tissue. The reason we did not see an interaction between N and P may be because the magnitudes of the N-P interactions were the same for both shoot and roots (i.e. the effect of N on biomass changed in a similar magnitude with respect to changing P in both roots and shoots), and so in calculating the shoot:root ratio the interaction simply cancelled out.

However, when comparing the responses of biomass, total C and total N to N fertilisation (**Figure 2**) and N:P ratio (**Figure 3**) between shoots and roots, differences were observed. The synergistic effect of N and P was higher in shoots compared to roots, and shoots appeared to respond more strongly to N than roots (**Figure 2**). The responses to N:P ratio (**Figure 3**) showed that when P was limiting, the magnitude of the responses to increased N was the same for shoots and roots (same magnitude slopes). Whereas when P was not limiting, the magnitude of the responses to N were suddenly much greater in shoots compared to roots (increased slopes). This could mean that when P was not limiting, relatively more resource was directed to shoots than roots in comparison to when P was limiting. Results to this effect have been reported by Hilbert (1990) and Scheible et al. (1997). But to our knowledge, this is the first study to investigate N-P interactions of this scale on root biomass and nutrient content.

### Nitrogen and phosphorus use efficiencies

Our calculations of NUE and PUE, were in essence a standardisation of the biomass to the amount of fertiliser applied. As a result, we compare how much biomass was produced per mmol N or P applied with increasing N and P. As expected, NUE decreased with increasing N fertilisation and PUE decreased with increasing P fertilisation, in both shoots and roots. In contrast, NUE and PUE increased with increasing P and N respectively. Duncan et al. (2018b) showed that NUE in wheat increased with addition of P, and also K, compared to just N alone and attributed this to increases in root mass and architecture. Similarly, Mehrparvar et al. (2021) showed that when sunflower plant’s P and K requirements were met, the required N fertilisation rate decreased whilst NUE increased. Our NUE data fit with these findings but there is little literature on the effect of N on PUE for comparison with our results. Similar results of N having positive effect on plant P dynamics, and vice versa, were observed in Yan et al. (2015). They showed that P increased N resorption efficiency and likewise, N increased P resorption efficiency.

A decrease in NUE due to decreased P availability could be explained by investments of N for P mining (i.e., production of phosphatase enzymes) which could trade-off against direct investment of N for growth, as was shown for microorganisms (Ramin and Allison, 2019). Similarly, Marklein and Houlton (2012) demonstrated that P-mineralizing phosphatase enzyme activity in roots increased with N fertilization under P limitation, and Fujita et al. (2010) showed that increased N fertilisation promoted phosphatase activity for improved P uptake.

We expected, as with previous results that there would be an interaction between N and P for NUE and PUE. This would mean that, for example, the rate of decrease in NUE due to increased N would change with increasing P, resulting in different slopes for the different P levels. We did not see this, as the relationship of NUE to N remained unchanged for all P levels in both shoots and roots. However, with PUE there was an N-P interaction in both roots and shoots, as the relationship of PUE to N was not the same for each P level. As explained above, PUE peaked with balanced N and P supply when P was low. But for the higher P levels, a different pattern emerged. We saw for the first time in this investigation a potential critical value of N for PUE in shoots where P > 0.25 mmol (**Figures 5B**-i). When P was not limiting, shoot PUE increased with increasing N fertilisation and peaked at N = 3.75 mmol. Above this level, PUE in shoots remained constant despite further increases in N. The saturation of PUE occurred at the N level containing 50% of the original Hoagland solution. Hoagland solution should contain the perfect amount of nutrients to enable a plant to grow, and yet 50% N was all that was required to reach the maximum PUE.

## Conclusions

Our data show two main findings: Firstly, there were positive synergistic N-P interaction effects on biomass, total C, and total N in both roots and shoots. And secondly, the synergistic effects of N and P were only observed above a critical level of P fertilisation (P ≥ 0.25 mmol). Below this critical level, N-P interactions were additive. We provide, for the first time, stoichiometric evidence that critical levels for synergistic interactions between N-P exist in barley and may exist in other crop plants, which could determine whether there is under- or overyielding of crop biomass or nutrient content.

There was no combined critical value of N and P, around which resource allocation shifted between shoots and roots, but solely a critical P value. In low P treatments (below the critical level), the plants did not show increases in biomass or nutrient content, even with ample N addition. The response of shoots and roots to N was the same below the critical P value, but above the critical value, shoots became more responsive to N and plants disproportionally increased their investment into shoots. Accordingly, the absolute amount of available P determined how the plant reacted to changes in N. This outcome could possibly change the way we interpret stoichiometry results, since usually stoichiometry deals with ratios, as a way to understand the ecological effects of relative changes in nutrients, but our study strongly suggests that we may need to identify key thresholds beyond which the system and the stoichiometric interaction functions quite differently.

## Data availability statement

The raw data supporting the conclusions of this article will be made available by the authors, without undue reservation.

## Author contributions

JC: Data curation, Formal analysis, Investigation, Project administration, Visualization, Writing – original draft, Writing – review & editing. KL: Conceptualization, Data curation, Methodology, Project administration, Writing – review & editing. MS: Data curation, Methodology, Writing – review & editing. VT: Conceptualization, Supervision, Writing – review & editing. MB: Conceptualization, Funding acquisition, Project administration, Resources, Supervision, Writing – review & editing.
