# Supplementary material for: Two-way NxP fertilisation experiment on barley (Hordeum vulgare) reveals shift from additive to synergistic N-P interactions at critical phosphorus fertilisation level
Source: Front Plant Sci. 2024 Mar 5;15:1346729. doi: 10.3389/fpls.2024.1346729 (PMC10948440; doi:10.3389/fpls.2024.1346729)
Supplement: Supplementary file 1 [file DataSheet_1.pdf]

Two-way NxP fertilisation experiment on barley (*Hordeum vulgare*) reveals shift from additive to synergistic N-P interactions at critical phosphorus fertilisation level

Jessica Clayton*[1], Kathleen Lemanski[2], Marcel Domink Solbach[3], Vicky M. Temperton[4], Michael Bonkowski*[5]

[1] Department Soil System Science, Helmholtz Centre for Environmental Research – UFZ, Halle (Saale), Germany
[2] Institut für Biologischen Pflanzenschutz, Julius Kühn-Institut, Dossenheim, Germany
[3] Department of Terrestrial Ecology, University of Cologne, Cologne, Germany
[4] Faculty of Sustainability, Leuphana University Lüneburg, Institute of Ecology, Lüneburg, Germany
***Correspondence:**
Jessica Clayton: jessica.clayton@ufz.de
Michael Bonkowski: m.bonkowski@uni-koeln.de

Photograph depicting germination method and experimental setup of pot experiment (Fig. S1).

Fig. S1 Experimental setup: (A) Seedlings for germination. (B) Final pot arrangement.

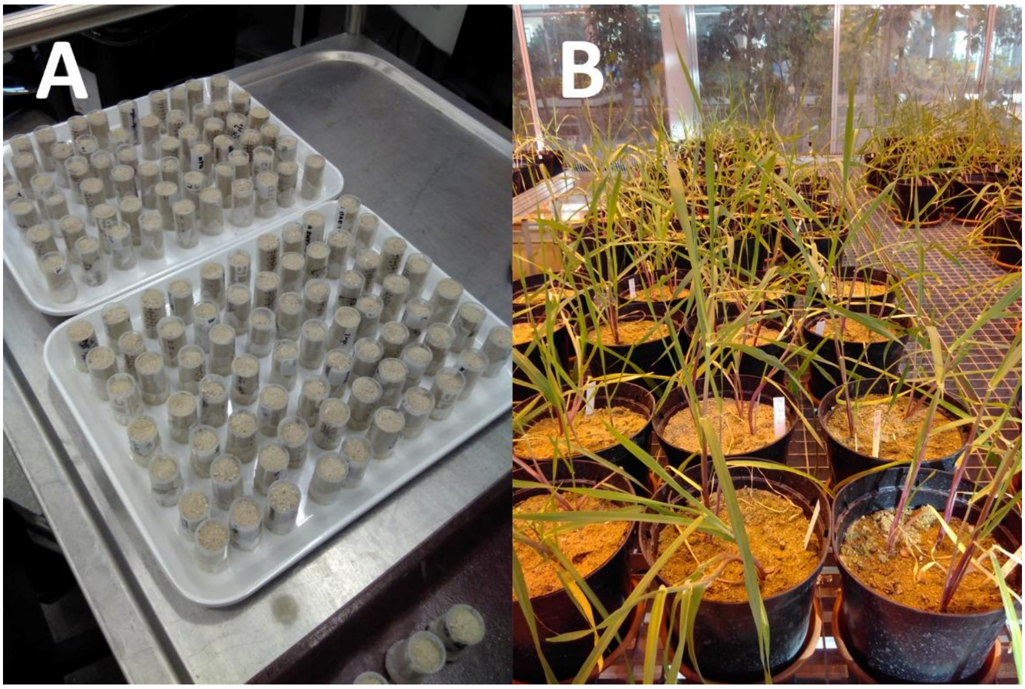

The supplementary information provided here gives information on the fertilisation solutions used (Table S1) and detailed statistical model outputs (Tables S2-S11).

Table S1. Table showing the recipes for fertilisation solutions of the 36 different treatments (N and P level are given in terms of percentage of the full Hoagland solution).

Treatment		Concentration of compounds (mmol/l)					
**N level (%)**	**P level (%)**	**KH_2PO_4**	**KNO_3**	**$Ca(NO_3)_2$**	**$MgSO_4$**	**KCl**	**CaCl**
0	0	0	0	0	2	6	5
12.5	0	0	0.625	0.625	2	5.375	4.375
25	0	0	1.25	1.25	2	4.75	3.75
50	0	0	2.5	2.5	2	3.5	2.5
75	0	0	3.75	3.75	2	2.25	1.25
100	0	0	5	5	2	1	0
0	12.5	0.125	0	0	2	5.875	5
12.5	12.5	0.125	0.625	0.625	2	5.25	4.375
25	12.5	0.125	1.25	1.25	2	4.625	3.75
50	12.5	0.125	2.5	2.5	2	3.375	2.5
75	12.5	0.125	3.75	3.75	2	2.125	1.25
100	12.5	0.125	5	5	2	0.875	0
0	25	0.25	0	0	2	5.75	5
12.5	25	0.25	0.625	0.625	2	5.125	4.375
25	25	0.25	1.25	1.25	2	4.5	3.75
50	25	0.25	2.5	2.5	2	3.25	2.5
75	25	0.25	3.75	3.75	2	2	1.25
100	25	0.25	5	5	2	0.75	0
0	50	0.5	0	0	2	5.5	5
12.5	50	0.5	0.625	0.625	2	4.875	4.375
25	50	0.5	1.25	1.25	2	4.25	3.75
50	50	0.5	2.5	2.5	2	3	2.5
75	50	0.5	3.75	3.75	2	1.75	1.25
100	50	0.5	5	5	2	0.5	0
0	75	0.75	0	0	2	5.25	5
12.5	75	0.75	0.625	0.625	2	4.625	4.375
25	75	0.75	1.25	1.25	2	4	3.75
50	75	0.75	2.5	2.5	2	2.75	2.5
75	75	0.75	3.75	3.75	2	1.5	1.25
100	75	0.75	5	5	2	0.25	0
0	100	1	0	0	2	5	5
12.5	100	1	0.625	0.625	2	4.375	4.375
25	100	1	1.25	1.25	2	3.75	3.75
50	100	1	2.5	2.5	2	2.5	2.5
75	100	1	3.75	3.75	2	1.25	1.25
100	100	1	5	5	2	0	0

Table S2. Manova model 1 summary (Response variables ~ N.fertilisation*P.fertilisation, n= 219).

Model term	Wilks λ	F statistic	df1	df2	p
N fertilisation	0.22	127.16	6	209	< 0.001
P fertilisation	0.26	99.01	6	209	< 0.001
NxP interaction	0.42	48.63	6	209	< 0.001

Table S3. Summary of the individual Anova analyses of each response variable from Manova model 1 (Response variable ~ N.fertilisation*P.fertilisation).

		Model term					
		N fertilisation		P fertilisation		NxP interaction	
**Response variable**	**Plant tissue**	**F statistic**	**p**	**F statistic**	**p**	**F statistic**	**p**
**Total biomass (g)**	root	63.75	< 0.001	188.04	< 0.001	94.73	< 0.001
	shoot	227.79	< 0.001	568.59	< 0.001	209.1	< 0.001
**Total C (mmol)**	root	83.66	< 0.001	253.63	< 0.001	129.14	< 0.001
	shoot	226.41	< 0.001	580.35	< 0.001	231.11	< 0.001
**Total N (mmol)**	root	280.54	< 0.001	128.2	< 0.001	142.75	< 0.001
	shoot	335.2	< 0.001	105.9	< 0.001	72.66	< 0.001

Table S4. Manova summary from model 2 (Response variables ~ log_{10}(N:P ratio)*P.fertilisation, n= 219).

Model term	Wilks λ	F statistic	df1	df2	p
**Log_{10}(N:P)**	0.41	33.62	6	143	< 0.001
**P fertilisation**	0.15	140.04	6	143	< 0.001
**Log_{10}(N:P)*P interaction**	0.29	57.29	6	143	< 0.001

Table S5. Summary of Anova analyses for each response variable of Manova model 2 (Response variables ~ log10(N:P ratio)*P.fertilisation).

		Model term					
		**N fertilisation**		**P fertilisation**		**NxP interaction**	
**Response variable**	**Plant tissue**	**F statistic**	**p**	**F statistic**	**p**	**F statistic**	**p**
**Total biomass**	Root	16.72	< 0.001	110.09	< 0.001	83.85	< 0.001
	Shoot	78.07	< 0.001	684.31	< 0.001	268.45	< 0.001
**Total C (µmol)**	Root	25.53	< 0.001	169.45	< 0.001	130.98	< 0.001
	Shoot	74.38	< 0.001	639.18	< 0.001	269.35	< 0.001
**Total N (µmol)**	Root	5.62	0.02	168.6	< 0.001	112.89	< 0.001
	Shoot	18.81	< 0.001	213.53	< 0.001	81.8	< 0.001

Table S6. Statistics of linear models giving slope (α) shown in Fig 3 for biomass, total C, total N in roots and shoots for each P fertilisation level (Linear model: Response variable ~ $\log_{10}$(N:P)).

Response variable	Tissue	P fertilisation (mmol)	Slope (α)	Standard error	T statistic	p
Biomass	Shoot	0.0625	-0.025	0.019	-1.283	0.21
		0.125	-0.004	0.034	-0.129	0.898
		0.25	0.291	0.051	5.749	< 0.001
		0.375	0.447	0.058	7.716	< 0.001
		0.5	0.622	0.05	12.332	< 0.001
Biomass	Root	0.0625	-0.06	0.019	-3.159	< 0.01
		0.125	-0.006	0.022	-0.277	0.784
		0.25	0.131	0.042	3.118	< 0.01
		0.375	0.115	0.049	2.326	0.028
		0.5	0.282	0.045	6.282	< 0.001
Total C	Shoot	0.0625	-1.352	0.663	-2.039	0.051
		0.125	-0.35	0.91	-0.384	0.703
		0.25	10.301	1.836	5.61	< 0.001
		0.375	16.27	1.87	8.699	< 0.001
		0.5	22.409	2.046	10.952	< 0.001
Total C	Root	0.0625	-1.813	0.575	-3.151	< 0.01
		0.125	-0.391	0.716	-0.546	0.589
		0.25	3.456	1.137	3.039	< 0.01
		0.375	4.819	1.131	4.26	< 0.001
		0.5	8.917	1.203	7.412	< 0.001
Total N	Shoot	0.0625	0.404	0.076	5.296	< 0.001
		0.125	0.73	0.078	9.355	< 0.001
		0.25	1.277	0.185	6.899	< 0.001
		0.375	1.293	0.24	5.38	< 0.001
		0.5	1.432	0.109	13.175	< 0.001
Total N	Root	0.0625	0.006	0.015	0.379	0.707
		0.125	0.069	0.026	2.65	0.013
		0.25	0.177	0.025	7.097	< 0.001
		0.375	0.223	0.028	7.878	< 0.001
		0.5	0.254	0.034	7.572	< 0.001

Table S7. Manova summary from model 3 (Shoot:root ratio (SR) of response variables ~ N.fertilisation*P.fertilisation, n=219).

Model term	Wilks λ	F stat	df1	df2	p
N fertilisation	0.77	20.84	3	212	< 0.001
P fertilisation	0.68	34.01	3	212	< 0.001
NxP interaction	0.95	3.58	3	212	0.015

Table S8. Summary of Anova analyses for each response variable of Manova model 3 (shoot:root ratio (SR) of response variables ~ N.fertilisation*P.fertilisation)

Response variable	N fertilisation		P fertilisation		NxP interaction	
	F statistic	p	F statistic	p	F statistic	p
SR-total biomass (g)	36.82	< 0.001	52.02	< 0.001	0.41	ns
SR-total C (mmol)	42.55	< 0.001	73.71	< 0.001	2.17	ns
SR-total N (mmol)	53.58	< 0.001	0.01	ns	2.7	ns

Table S9. Manova summary for each response variable (nitrogen-use efficiency (NUE) and phosphorus-use efficiency (PUE) in shoots and roots, i.e., Shoot-NUE, Shoot-PUE, Root-NUE and Root-PUE) of model 4 (Response variables ~ N.fertilisation*P.fertilisation, n=219).

Model term	df	Wilks λ	F statistic	df1	df2	p
N fertilisation	1	0.23	121.2	4	147	< 0.001
P fertilisation	1	0.21	136.15	4	147	< 0.001
NxP interaction	1	0.82	8.11	4	147	< 0.001

Table S10. Summary of Anova analyses for each response variable (nitrogen-use efficiency (NUE) and phosphorus-use efficiency (PUE) in shoots and roots) of Manova model 4 (Response variables ~ N.fertilisation*P.fertilisation).

Tissue	Nutrient-use efficiency	N fertilisation		P fertilisation		NxP interaction	
		F statistic	p	F statistic	p	F statistic	p
Root	NUE	191.61	< 0.001	15.17	< 0.001	0.01	ns
	PUE	2.12	ns	248.55	< 0.001	23.13	< 0.001
Shoot	NUE	428.62	< 0.001	94.28	< 0.001	2.22	ns
	PUE	31.93	< 0.001	412.16	< 0.001	23.86	< 0.001

Table S11. Min, max and range of N:P ratio values for each P fertilisation level (P > 0).

P fertilisation (mmol)	N:P min	N:P max	Range
0.0625	15	120	105
0.125	7.5	60	52.5
0.25	3.75	30	26.25
0.375	2.5	20	17.5
0.5	1.875	15	13.125
